# Interannual variation in food choice of white‐headed langur inhabiting limestone forests in Fusui, southwest Guangxi, China

**DOI:** 10.1002/ece3.7726

**Published:** 2021-06-12

**Authors:** Shiyi Lu, Ting Chen, Zhonghao Huang, Youbang Li, Changhu Lu

**Affiliations:** ^1^ College of Biology and the Environment Nanjing Forestry University Nanjing China; ^2^ Guangxi Forest Resources and Environment Monitoring Center Nanning China; ^3^ Observation and Study Station of National Forest Ecosystem in Guangxi Dayaoshan Laibin China; ^4^ College of Life Sciences Guangxi Normal University Guilin China

**Keywords:** food choice, interannual variation, limestone forest, white‐headed langurs

## Abstract

Food habits are important factors in the adaptation of wild nonhuman primates. White‐headed langurs (*Trachypithecus leucocephalus*) are endemic to heavily fragmented limestone forests and adapt to unique living environments via flexible food selection strategies. In this study, we compared the dietary data for white‐headed langurs living in Chongzuo White‐headed Langur National Nature Reserve in 2013 and 2016 to evaluate interannual variations in diet. Our results indicated that young leaves were the main food source for langurs, accounting for 52.4% (*SD* 25.4%) and 65.2% (*SD* 22.4%) of their diet in 2013 and 2016, respectively. The pattern of plant part consumption was similar between the two years. The consumption of young leaves varied with the availability of young leaves, whereas the consumption of mature leaves was negatively correlated with young leaf availability. The consumption of plant species and diet diversity were higher in 2013 than in 2016. In both 2013 and 2016, although diet diversity varied with the consumption of mature leaves, it was negatively correlated with the consumption and availability of young leaves. Dietary interannual variation is likely to either be linked to phenological variations or indicate that white‐headed langurs have a flexible ecological adaptation coping with habitat fragmentation.

## Graphical abstract

This manuscript for the first time considers the interannual variation of white‐headed langur in limestone forest and found that the plant species and diet diversity varied between 2013 and 2016. Dietary interannual variation is likely to be linked to the phenology variations or mean that the white‐headed langurs has a flexible ecological adaptation coping with habitat fragmentation.

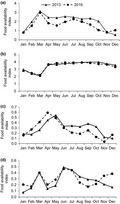

## 1 INTRODUCTION

Feeding accounts for the largest proportion of the daily activities of most primates, including food choices, movement between food patches, and feeding time budget (Fleagle, 2013). The feeding strategy is an important factor for the adaptation of wild nonhuman primates (Huang et al., 2008). Most wild primates adjust their food composition according to seasonal fluctuations in food availability in the environment (Richard, 1985). For example, limestone‐living rhesus macaques (*Macaca mulatta*) increase their consumption of fruit during the rainy season when fruits are abundant (Tang et al., 2016). The food composition of the sympatric François’ langur (*Trachypithecus francoisi*) and Assamese macaques (*Macaca assamensis*) varies seasonally, gradually separating their dietary niche to decrease interspecific competition (Zhou et al., 2018). Moreover, interannual variation in food availability also affects the feeding strategy of wild primates (Cui et al., 2020; Hill & Agetsuma, 1995; Zhou et al., 2009). Especially in seasonal food items, such as fruits and seeds, the yield is higher in a mast year and is scarce in non‐mast years, with a pattern of alternating between the mast years and non‐mast years (Cui et al., 2020; Hill & Agetsuma, 1995). For instance, Taihangshan macaques (*Macaca mulatta tcheliensis*) consumed more leaves in the non‐mast years when seeds were not available (Cui et al., 2020), while Japanese macaques (*Macaca fuscata yukui*) consumed more fruits and decreased feeding time in leaves in the heavy‐fruiting years (Hill & Agetsuma, 1995). Therefore, the study of feeding behavior and variation makes an important contribution to our understanding of the environmental adaptation of wild primates.

Habitat loss and fragmentation are the greatest threats to animal protection, especially for species living in highly fragmented forest habitats (Chaves et al., 2012). Habitat fragmentation usually changes the composition and structure of forest vegetation, decreasing habitat quality and plant species number and diversity, and changing both the food distribution patterns and the quantity of food available (Arroyo‐Rodríguez & Mandujano, 2006; Chiarello & Marsh, 2003; Fahrig, 2003). In fragmented habitats, the survival of primates depends greatly on their ability to respond to environmental changes, such as their behavioral activities and the plasticity of eating habits (Wong & Sicotte, 2007), including increasing the type of food consumed to broaden the diet, increasing the proportion of low‐quality foods (mature leaves), and using more vines and secondary successional species (Hu, 2011; Soule, 1986; Zhou et al., 2009). For example, spider monkeys (*Ateles geoffroyi*) in the fragmented forest habitats of the south of Mexico diversify their overall diet and consume more leaves (both mature leaves and young leaves) and non‐tree growth forms than those living in continuous forests (Chaves et al., 2012). Similarly, *Alouatta pigra* in the Kharak Mo area of Mexico adopt similar strategies to cope with habitat fragmentation (Rivera & Calmé, 2006). In the fragmented limestone forest, François’ langurs eat more vines (Li et al., 2012).

White‐headed langur (*T*. *leucocephalus*) is a critically endangered primate species endemic to China. It is confined to a narrow triangular limestone hill in the four counties of Longzhou, Ningming, Jiangzhou, and Fusui, in southwest Guangxi Province, with a total habitat of approximately 200 km^2^ (Huang, 2002). Limestone hills are vulnerable to human disturbance, which leads to habitat fragmentation. Habitat fragmentation decreases vegetation biomass and plant progeny quality, heavily negatively affecting the food resources of herbivores (Aguilar et al., 2019), which in turn forces herbivores to develop foraging strategies that differ from other populations living in continuous habitats. Compared with populations inhabiting the lightly fragmented Fusui forests, white‐headed langurs living in the more heavily fragmented Chongzuo forests spend more time feeding and less time moving, with a smaller home range and consuming more species of plants (Zhang et al., 2017). Moreover, as an adaptation strategy to fragmented habitats, white‐headed langurs choose food species according to their dominance in the habitat to improve feeding efficiency and minimize the risk of predation (Zheng et al., 2020). Furthermore, most likely due to seasonal variations in food availability, the food diversity and composition of white‐headed langurs vary seasonally. Langurs have been found to significantly increase their food diversity, consuming more young leaves and less mature leaves, in the rainy season than in the dry season (Huang et al., 2000; Huang, Wu, et al., 2008; Li & Rogers, 2006; Li et al., 2003; Yin et al., 2011; Zhou et al., 2013). However, previous studies are limited to a duration of ≤1 year, and variations in the seasonal and annual level of food resources in the environment are likely to exist due to seasonal and interannual phenological changes (Zhou et al., 2009). In addition, fragmented limestone forests suffer from severe edge effects that create instability, resulting in the food sources of white‐headed langurs being susceptible to change (Huang, Li, et al., 2008). Therefore, studies on the diet of white‐headed langurs in limestone habitats over the long term are necessary not only to explore their diet patterns and food choice strategies, but also to understand the mechanisms underlying the adaptation of primates to habitat fragmentation.

In this study, we examined interannual variations in the dietary composition of the same white‐headed langur group in the Chongzuo White‐headed Langur National Nature Reserve during two separate study periods. We collected data on the diet of white‐headed langurs to evaluate the feeding strategy that the animals adopted in the fragmented limestone forests. The aim of this study was to identify the interannual variations in food types, species composition, and food diversity of white‐headed langurs and the extent of dietary similarity between two years.

## 2 METHODS

### 2.1 Study site and subjects

The study site was located in the Chongzuo White‐headed Langur National Nature Reserve in southwest Guangxi Province, China (22°24–22°46N, 107°22–107°42E). The reserve is characterized by limestone seasonal rainforests with hills ranging from 400 to 600 m above sea level (Tan, 2014). The habitats of white‐headed langurs are roughly divided into flat land, cliffs, and hilltops. As a result of long‐term human activities, flat lands among the hills have been converted to farmland, decreasing the continuity between hills. From 1973 to 1999, the proportion of forests to total area decreased from 62.78% to 43.13%; the area of farmland, however, increased from 28.89% to 38.77%; the number of agricultural patches increased from 48 to 107, and the fragmentation index increased from 2.30 to 5.23 (Chen et al., 2008). The reserve is partly surrounded by large‐scale agricultural sugarcane plantations; some hills in this area are completely isolated (Huang, 2002; Huang et al., 2007), and the vegetation is severely disturbed ( Li & Rogers, 2004, 2005).

This study was carried out during two separate periods: January to December 2013 and January to December 2016. We collected climatic data, including minimum and maximum temperatures, using a thermometer (MX2301; HOBO), and rainfall using a rain gauge (MX2301; HOBO). The average annual rainfall was 977 mm in 2013 and 1,022 mm in 2016. Following Zhou et al. (2009), two distinct seasons were identified: a rainy season from April to September with >50 mm monthly rainfall and a dry season in the remainder of the year with <50 mm monthly rainfall.

The primates in the study group were semi‐habituated to observers and were able to tolerate observers within a distance of 20 m. The study group initially consisted of 19 individuals (one adult male, nine adult females, and nine immatures). The group size increased to 22 following the birth of three infants in 2013. In 2016, when observation began, the group was composed of 15 individuals (one adult male, eight adult females, and six immatures) and increased to 18 individuals with the birth of three infants by the end of the study period. Since newborns born between April and July (this study; Huang, 2002; We, 2014) did not feed on plants, they were excluded throughout the study period.

### 2.2 Data collection

Due to the topographical conditions of limestone habitats (sharp rocks and steep cliffs), the observers were unable to follow the white‐headed langur; they observed the langurs from the cultivated flatland surrounding the fragmented habitats. The primates usually left their sleeping site in the early morning around 6:00 a.m. during the rainy season and 7:00 a.m. in the dry season (Huang et al., 2006). Accordingly, during each full day of observation, the behavioral observation of the langurs was initiated after locating the primates near their sleeping sites at dawn and finalized at nightfall, without ever losing contact with the animals for more than 30 min. During each partial day of observation, data collection was started whenever we located the langurs and finalized if contact was lost for more than 30 min or the animals entered a sleeping site. The majority of the partial days of observations occurred when the langurs moved to the hilltops for resting at noon. Instantaneous scan sampling (Altmann, 1974) was used at 15‐min intervals. The scans lasted for 5 min, followed by 10 min of inactivity until the next scan began. The activity of each individual was recorded during each scan. Each individual was observed for 5 s after detection, and its predominant behavior during that interval was recorded. To avoid sampling bias toward certain individuals or a particular age–sex class, the behavioral records of as many different individuals as possible during a scan were collected to ensure that all individuals in the focal group were included; no individual was sampled more than once during a scan. When an individual was feeding, the plant species and parts eaten were recorded (e.g., mature leaves, young leaves, fruits, flowers, seeds, and petioles). The food species of the white‐headed langur have long been studied (e.g., Huang, 2002; Huang et al., 2000; Huang, Wu, et al., 2008), and a reference book of their preferred food species is currently being drafted by our research group (unpublished). The majority of the time when the langurs were feeding their food being used could be easily observed using a high‐quality telescope (Nikon Fieldscope ED82; 25‐75X Zoom, Japan), using the reference book to identify the food species. When the maturity of some leaves was unknown, the plant parts were recorded as leaves of unknown maturity. When some species could not be identified, we recorded the entry as the most plant species eaten and collected specimens for subsequent identification. In total, 2,668 feeding records were collected in 2013, 2,528 of which were identified as food items. In 2016, 2,599 feeding records were collected for the same group, 2,563 of which were identified as food items.

We conducted vegetation surveys at the study site at the onset of the behavioral data collection. A stratified random sampling method was used for the placement of the vegetation plots. We placed 13 plots (50 × 10 m) in the home range of the animals, including four in the valley basins and nine on the hillsides. The plots covered three vegetation types, including arbor, shrub, and lianas. Grass was excluded as langurs were never observed to feed on it. Within the plots, all trees with a diameter at breast height (DBH) ≥5 cm were measured and tagged. We determined the limit of 5 cm from a pilot observation, which showed that most foraging by langurs occurred in trees of this size and larger. We visually inspected all tagged trees (*n* = 312) within the vegetation plots in both 2013 and 2016 for the presence of young leaves, fruits, and flowers on a monthly basis to evaluate seasonal and interannual variations in plant part availability. We surveyed the phenological variation of all the marked trees in the vegetation sample monthly and recorded the development of young leaves, mature leaves, flowers, and fruits (Huang et al., 2010; Tang et al., 2016). We used the method of Tweheyo et al. (2004) to estimate plant part scores by classifying the cover of branches into five categories: ≤10% cover (a tree with leaves that cover less than 10% of the branches); 10% and <25% cover; 25% and <50% cover; 50% and <75% cover; and 75% cover.

### 2.3 Data analysis

Following Heiduck (1997), we averaged the score across all marked trees in the phenological sample to represent the food availability index. We determined the percentage of different plant species in the diet by calculating the percentage of feeding records devoted to them among the annual total feeding records. Food category composition was expressed as the percentage of different plant parts in the monthly diet of the study group using monthly total feeding records. The annual consumption of plant species was determined by averaging the monthly percentages. We calculated the Shannon–Wiener diversity indices to represent the diversity of plant species consumption. The Shannon–Weaver diversity index was calculated using the following equation:

$$H_{\mathrm{shannon}}=‐\sum_{i=1}^{S}\frac{N_{i}}{N}\ln\frac{N_{i}}{N}$$

where *N_i_* is the number of feeding records of the *i*th plant species, and *N* is the sum of the feeding records of the plant species. Annual differences in food habits were determined using the overlap index (OI) (Whittaker & Fairbanks, 1958), which was calculated for all yearly combinations for each month. The OI varies from 0 (completely different) to 100 (complete overlap) and is defined by the following equation:

$$OI=\left(1‐\frac{1}{2}\sum \left|P_{a}‐P_{b}\right|\right)\times 100.$$

where *P_a_* and *P_b_* are the feeding proportions (from 0 to 1) of a particular food item or category in years a and b, respectively. We calculated the OI separately for foods at the item and species levels.

Data collected in the partial‐day observations consisted of less than 1% of the total data. Thus, we excluded records collected in partial‐day observations, and only full‐day observations were included in the analysis. Full‐observation days varied among months and years; thus, we used a nonparametric test because the sample size was small and somewhat unbalanced (full‐day observation days ranged from 3 to 10 days) (Table 1). The Wilcoxon signed‐rank test was used to examine interannual variations in the overall pattern of use of different plant species and parts. We used the Mann–Whitney *U* test to compare the monthly averages of the percentage of feeding records for various food items from the rainy and dry seasons. Spearman rank correlation was used to test the relationship between the abundance and consumption of different plant species and plant parts. All tests were performed using SPSS 23.0 with a significance level of 0.05.

**TABLE 1:** Monthly percentage of the feeding of different plant parts in Chongzuo White‐headed Langur National Nature Reserve in 2013 and 2016

Months	Sample days	Leaves	Young leaves	Mature leaves	Leaves unknown maturity	Fruits	Young fruits	Mature fruits	Fruits unknown maturity	Stems	Seeds	Flowers	Petioles	Others
2013														
Jan	8	86.3	29.0	54.1	3.3	2.2	0.8	0.3	1.1	7.7	0.3	3.0	0.5	0
Mar	9	75.9	73.0	1.9	1.0	2.4	1.1	1.3	0	5.8	1.1	14.9	0	0
Apr	10	84.0	77.8	5.2	1.0	10.3	9.9	0.4	0	1.6	0.4	3.7	0	0
May	4	92.7	85.8	6.7	0.2	2.3	2.2	0.1	0	2.7	0	2.0	0	0.3
Jun	7	78.0	75.3	0.7	2.0	7.6	5.9	0	1.7	2.5	0	3.9	0	8.0
Jul	7	31.8	30.4	0.8	0.6	52.9	51.2	1.7	0	5.4	6.8	3.0	0	0.2
Aug	6	61.5	59.7	1.4	0.4	33.2	29.9	2.1	1.3	2.7	0.3	2.1	0	0.3
Sep	5	63.6	59.3	3.7	0.6	9.2	8.7	0.2	0.3	6.6	4.1	16.5	0	0
Oct	6	63.2	53.3	9.7	0.1	12.9	9.2	3.6	0.1	13.7	2.9	7.2	0.1	0
Nov	6	46.3	21.0	24.7	0.6	8.4	7.1	1.1	0.1	21.0	9.2	14.8	0.3	0
Dec	5	67.5	11.9	55.6	0	1.1	0.3	0.5	0.3	24.3	4.7	2.1	0	0.2
Annual Mean		68.3	52.4	14.9	0.9	12.9	11.5	1.0	0.5	8.5	2.7	6.6	0.1	0.8
Annual *SD*		18.0	25.4	20.9	1.0	16.0	15.5	1.1	0.6	7.8	3.2	5.8	0.2	2.4
Dry season Mean		67.8	37.6	29.2	1.0	5.4	3.7	1.4	0.3	14.5	3.7	8.4	0.2	0
Dry season *SD*		14.9	25	24.8	1.3	5.1	4.1	1.3	0.4	8.1	3.6	6.2	0.2	0.1
Rainy season Mean		68.6	64.7	3.1	0.8	19.2	18	0.7	0.5	3.6	1.9	5.2	0	1.5
Rainy season *SD*		21.6	19.8	2.5	0.7	19.6	18.9	0.9	0.8	2.0	2.9	5.6	0	3.2
2016														
Jan	4	83.9	23.3	60.6	0	1.4	1.4	0	0	9.6	4.8	0.3	0	0
Mar	5	92.9	90.5	2.2	0.2	2.4	2.4	0	0	0.2	2.0	2.6	0	0
Apr	5	66.0	59.1	6.8	0	30.1	23.4	6.6	0	0.4	0.4	2.9	0	0.2
May	4	94.5	94.5	0	0	2.9	2.9	0	0	1.6	0	1.0	0	0
Jun	5	87.3	87.3	0	0	7.6	7.6	0	0	0.2	0	1.7	0	3.2
Jul	5	82.4	81.1	1.3	0	14.4	14.1	0.4	0	0.9	0.8	1.5	0	0
Aug	5	78.5	72.0	6.5	0	21.0	20.5	0.5	0	0.5	0	0	0	0
Sep	4	69.9	64.2	5.6	0	23.4	23.4	0	0	0.3	0.5	5.9	0	0
Oct	5	56.1	49.5	6.6	0	26.4	18.4	8.0	0	0	17.5	0	0	0
Nov	5	74.5	54.7	19.8	0	4.1	1.0	3.1	0	3.6	12.6	5.3	0	0
Dec	3	64.2	41.5	22.7	0	2.2	2.2	0	0	15.7	14.4	2.6	0	0.9
Annual Mean		77.3	65.2	12.0	0	12.3	10.7	1.7	0	3.0	4.8	2.2	0	0.4
Annual *SD*		12.3	22.4	17.8	0.1	11.0	9.4	2.9	0	5.1	6.7	2.0	0	1.0
Dry season Mean		74.3	51.9	22.4	0	7.3	5.1	2.2	0	5.8	10.3	2.2	0	0.2
Dry season *SD*		14.7	24.6	23.0	0.1	10.7	7.5	3.5	0	6.8	6.6	2.1	0	0.4
Rainy season Mean		79.8	76.4	3.4	0	16.6	15.3	1.2	0	0.7	0.3	2.2	0	0.6
Rainy season *SD*		10.7	13.7	3.3	0	10.2	8.7	2.6	0	0.5	0.3	2.1	0	1.3

## 3 RESULTS

### 3.1 Food availability at the study site

**FIGURE 1:**
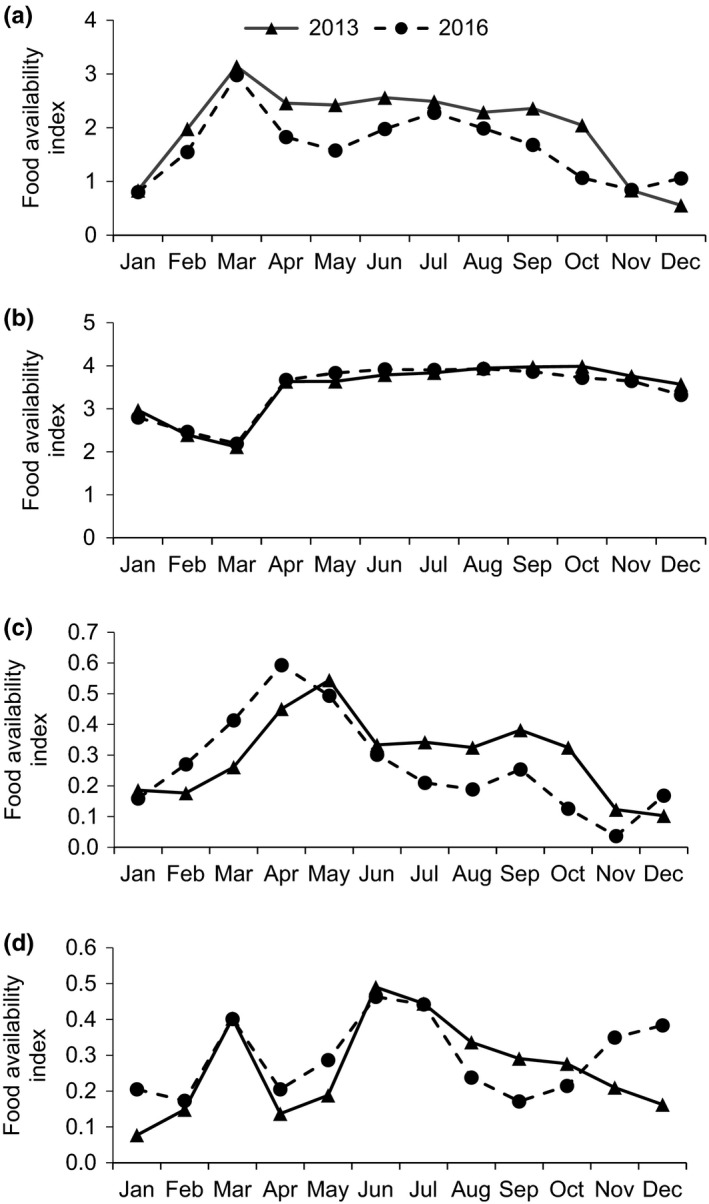
Monthly abundance of young leaves (a), mature leaves (b), fruits (c), and flowers (d) in Fusui study site in 2013 and 2016

In the habitat of white‐headed langurs, plant part availability showed a similar monthly trend during the two study periods. In both years, the highest availability of young leaves was observed in March, when mature leaves were lacking. However, the overall availability of young leaves was significantly higher in 2013 than in 2016 (*Z* = −2.312, *n* = 11, *p* =.020; Figure 1a). By contrast, mature leaves, flowers, and fruits did not show a marked interannual variation between the two years (mature leaves: *Z* = −0.711, *n* = 11, *p* = .477; flowers: *Z* = −0.889, *n* = 11, *p* = .374; fruits: *Z* = −0.800, *n* = 11, *p* = .424; Figure 1b, c, d).

In 2013, flowers were found to be more available in the rainy season than in the dry season (*Z* = −2.653, *n* = 11, *p* = .008), while the availability of other plant parts did not show significant seasonal variation (young leaves: *Z* = −1.643, *n* = 11, *p* = .100; mature leaves: *Z* = −1.278, *n* = 11, *p* = .201; fruits: *Z* = −1.095, *n* = 11, *p* = .273). In 2016, mature leaves and flowers were significantly more abundant in the rainy season than in the dry season (mature leaves: *Z* = −2.556, *n* = 11, *p* = .011; flowers: *Z* = −2.008, *n* = 11, *p* = .045), and the availability of young leaves and fruits did not show significant seasonal variation (young leaves: *Z* = −1.643, *n* = 11, *p* = .100; fruits: *Z* = −0.091, *n* = 11, *p* = .927).

### 3.2 Plant part consumption of white‐headed langurs

**FIGURE 2:**
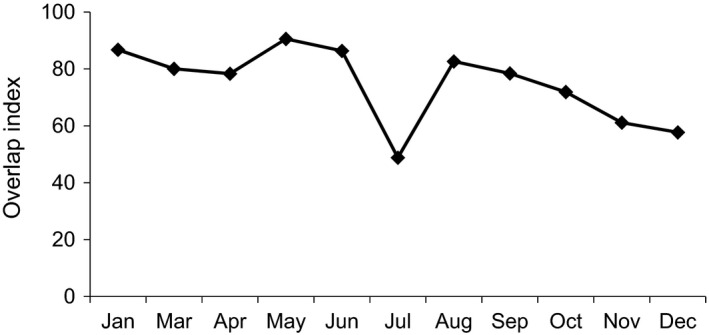
Seasonal changes in plant part overlap index (OI) values for foods eaten by White‐headed langurs in 2013 and 2016

The main source of food for white‐headed langurs in both 2013 and 2016 was leaves, accounting for 68.3% (*SD* 18.0%) and 77.3% (*SD* 12.3%), respectively, of which young leaves were the majority, accounting for 52.4% (*SD* 25.4%) in 2013 and 65.2% (*SD* 22.4%) in 2016. In 2013, in terms of preference of consumption of plant parts”, young leaves were followed by mature leaves, fruits, stems, flowers, seeds, others, and petioles. In 2016, the consumption of fruits was higher than that of mature leaves, followed by seeds, stems, and flowers. Petioles were not recorded in 2016 (Table 1). The consumption of young leaves, mature leaves, young fruits, mature fruits, seeds, petioles, and others was not significantly different between 2013 and 2016. The interannual variations are mainly reflected in the unknown maturity of leaves, fruits of unknown maturity, flowers, and stems (Table 2). The overlap index of food items in both years ranged from 48.81 to 90.59, where the highest value appeared in May and the lowest in July (Figure 2), with values greater than 70% for a total of 8 months. In summary, the white‐headed langurs were found to have similar food item compositions during the two study periods (2013 and 2016).

**TABLE 2:** Interannual and seasonal variation of plant parts consumed by white‐headed langurs in 2013 and 2016 (Wilcoxon signed‐rank test for interannual variation, *n* = 11; Mann–Whitney *U* test for seasonal variation, *n* = 11)

Variation		Leaves	Young leaves	Mature leaves	Leaves unknown maturity	Fruits	Young fruits	Mature fruits	Fruits unknown maturity	Stems	Seeds	Flowers	Petioles	Others
Annual		*Z* = −1.245	−1.867	−0.356	−2.803	−0.178	−0.711	−0.051	−2.366	−2.667	−0.889	−2.845	−1.604	−0.734
		*p* = .213	0.062	0.722	0.005	0.859	0.477	0.959	0.018	0.008	0.374	0.004	0.109	0.463
Seasonal	2013	*U* = 14.0	4.0	3.0	14.0	7.0	5.0	9.0	14.5	1.0	8.0	11.0	6.0	7.0
	2016	*p* = .855	0.045	0.028	0.855	0.144	0.068	0.273	0.926	0.011	0.200	0.465	0.037	0.111
		*U* = 11.0	5.0	4.0	12.0	6.0	3.0	14.5		12.0	‐	14.5	15.0	13.0
		*p* = .465	0.068	0.044	0.273	0.100	0.028	0.921	‐	0.584	0.006	0.927	‐	0.642

Food consumption showed significant seasonal differences. In 2013, the seasonal variation was marked in young leaves, mature leaves, stems, and petioles; the other food items did not show significant seasonal differences. The consumption of young leaves was significantly higher in the rainy season than in the dry season, in contrast to the seasonal variation of mature leaves, stems, and petioles (Table 2). In 2016, significant seasonal variation was observed in mature leaves, young fruits, and seeds. The mature leaves and seeds were consumed more in the dry season than in the rainy season, whereas the young fruits were consumed more in the rainy season. No significant differences were observed for the other food items (Table 2).

In this study, a correlation between the food item consumption of white‐headed langurs and food availability was observed (Table 3). The consumption of young leaves was significantly positively correlated with the availability of young leaves and flowers, and negatively correlated with mature leaf availability in both years. In 2013, the consumption of mature leaves was markedly negatively correlated with the availability of fruits; the consumption of fruits and young fruits was positively correlated with the availability of mature leaves, and the consumption of stems was negatively correlated with the availability of young leaves and flowers. In 2016, the feeding proportion of seeds was significantly negatively correlated with the availability of mature leaves and flowers.

**TABLE 3:** Spearman rank correlation analysis between the consumption and the availability of different plant parts (*n* = 11)

Availability	Leaves	Young leaves	Mature leaves	Leaves unknown maturity	Fruits	Young fruits	Mature fruits	Fruits unknown maturity	Stems	Seeds	Flowers	Petioles	Others
2013													
Young leaves	*r* = .109	0.718	−0.836	0.345	0.300	0.300	−0.145	−0.386	−0.736	−0.305	0.273	−0.595	0.188
	*p* = .750	0.013	0.001	0.298	0.370	0.370	0.670	0.241	0.01	0.361	0.417	0.053	0.579
Mature leaves	*r* = −.573	0.009	−0.345	−0.382	0.727	0.700	0.273	0.223	−0.027	0.118	0.173	−0.040	0.109
	*p* = .066	0.979	0.298	0.247	0.011	0.016	0.417	0.509	0.937	0.729	0.612	0.906	0.750
Flowers	*r* = .278	0.784	−0.506	0.036	0.387	0.492	−0.351	−0.378	−0.779	−0.411	−0.082	−0.507	0.216
	*p* = .408	0.004	0.113	0.915	0.239	0.124	0.290	0.252	0.005	0.209	0.811	0.111	0.523
Fruits	*r* = −.473	0.209	−0.855	0.009	0.464	0.382	0.173	0.098	−0.273	−0.014	0.327	−0.439	0.357
	*p* = .142	0.537	0.001	0.979	0.151	0.247	0.612	0.775	0.417	0.968	0.326	0.176	0.281
2016													
Young leaves	*r* = .345	0.745	−0.688	0.500	0.318	0.491	−0.03	‐	−0.564	−0.523	−0.050	‐	0.012
	*p* = .298	0.008	0.019	0.117	0.340	0.125	0.931	‐	0.071	0.099	0.884	‐	0.973
Mature leaves	*r* = .045	0.409	−0.579	−0.500	0.555	0.591	0.154	‐	−0.245	−0.651	−0.251	‐	0.058
	*p* = .894	0.212	0.062	0.117	0.077	0.056	0.652	‐	0.467	0.030	0.457	‐	0.866
Flowers	*r* = .436	0.700	−0.601	0.300	0.191	0.464	−0.292	‐	−0.309	−0.706	0.205	‐	0.283
	*p* = .180	0.016	0.050	0.370	0.574	0.151	0.383	‐	0.355	0.015	0.545	‐	0.399
Fruits	*r* = .436	0.445	−0.451	0.300	−0.400	−0.436	−0.258	‐	0.018	−0.046	−0.068	‐	0.295
	*p* = .180	0.170	0.164	0.370	0.223	0.180	0.444	‐	0.958	0.893	0.842	‐	0.379

### 3.3 Plant species diversity and composition consumed by white‐headed langurs

**FIGURE 3:**
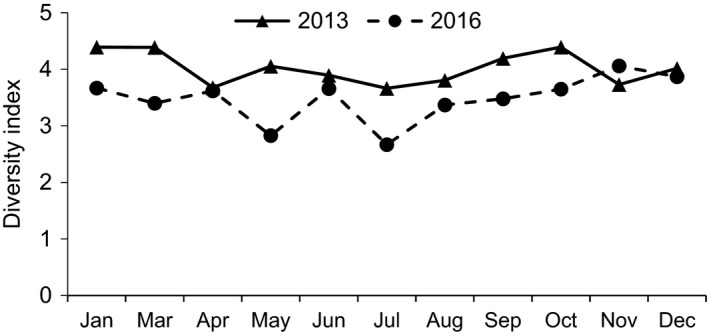
Monthly changes in diversity of plant species consumed by white‐headed langurs in 2013 and 2016

Food diversity showed significant interannual differences (Figure 3). The food diversity of 2013 (average 4.02 ± 0.28) was significantly higher than that of 2016 (average 3.48 ± 0.39) (*Z* = ‒2.578, *n* = 11, *p* = .010). No significant seasonal difference was observed for food diversity in 2013 (*Z* = ‒1.643, *n* = 11, *p* = .100), whereas in 2016, food diversity was significantly higher during the dry season than during the rainy season (*Z* = ‒2.008, *n* = 11, *p* = .045).

**FIGURE 4:**
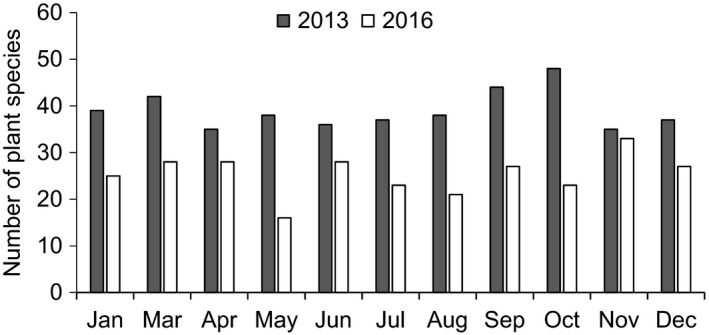
Number of plant species consumed by white‐headed langurs in 2013 and 2016

**FIGURE 5:**
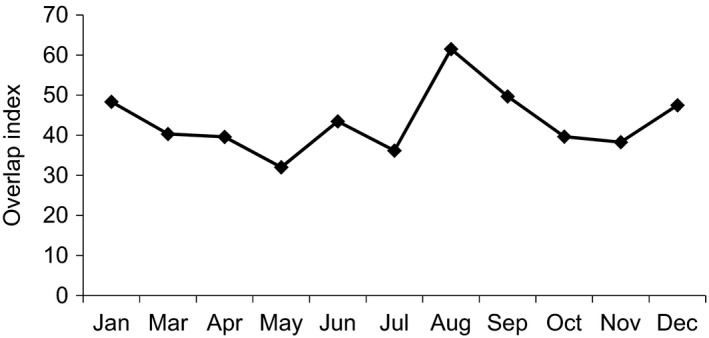
Seasonal changes in plant species overlap index (OI) values for foods eaten by white‐headed langurs in 2013 and 2016

The diets of langurs included a considerable number of species. They consumed 93 plant species in 2013 and 70 species in 2016 (Figure 4), of which 60 species were consumed in both years, accounting for 64.52% of the total food species in 2013 and 85.71% of that in 2016. The number of plant species consumed in 2013 was significantly higher than that in 2016 (*Z* = −2.941, *n* = 11, *p* = .003). However, no marked seasonal difference in the number of plant species consumed was found during the two study periods (2013: *Z* = ‒0.735, *n* = 11, *p* = .462; 2016: *Z* = ‒0.926, *n* = 11, *p* = .355). The overlap of plant species between the two periods peaked in August at 61.50% and was lowest in May at 32.03% (Figure 5).

Correlation analysis indicated that the number of plant species consumed and food diversity did not show a significant correlation with plant part availability in 2013 (Table 4). However, in 2016, the number of plant species was positively correlated with the consumption of flowers, and food diversity was positively correlated with the consumption of mature leaves and negatively correlated with the consumption and availability of young leaves (Table 4).

**TABLE 4:** Spearman rank correlation analysis between the number of plant species and the consumption and availability of plant parts, and the relationship between the food diversity and the consumption and availability of plant parts

	Number of plant species consumed		2016		Food diversity		2016	
	2013				2013			
	*r*	*p*	*r*	*p*	*r*	*p*	*r*	*p*
Consumption of plant part								
Leaves	.037	.915	−.120	.726	.427	.190	−.409	.212
Young leaves	−.005	.989	−.143	.675	−.064	.853	−.718	.013
Mature leaves	.064	.852	.236	.486	.391	.235	.692	.018
Leaves unknown maturity	−.252	.455	.304	.363	.018	.958	−.200	.555
Fruits	−.005	.989	−.046	.893	−.500	.117	−.291	.385
Young fruits	−.114	.738	−.240	.478	−.591	.056	−.518	.102
Mature fruits	.233	.490	.0150	.965	−.100	.770	.040	.908
Fruits unknown maturity	.068	.843	‐	‐	.228	.500	‐	‐
Stems	.284	.398	−.106	.756	.382	.247	.300	.370
Seeds	−.089	.794	.214	.528	−.246	.466	.560	.073
Flowers	.211	.534	.741	.009	.164	.631	.310	.354
Petioles	.125	.714	‐	‐	.393	.232	‐	‐
Others	−.284	.397	.419	.199	−.292	.383	.405	.217
Availability of plant part								
Young leaves	−.069	.841	−.041	.904	−.255	.450	−.727	.011
Mature leaves	.243	.472	−.415	.205	−.155	.650	−.473	.142
Flowers	.011	.973	.014	.968	−.237	.483	−.564	.071
Fruits	.082	.810	.161	.636	−.236	.484	−.055	.873

The animals foraged similar species during the two study periods. There was also a marked similarity in the percentages of feeding records for plant species between the two study periods. Among the top 10 food species, six species (*Pteroceltis tatarinowii*, *Canthium dicoccum*, *Cansjera rheedei*, *Sterculia monosperma*, *Ventilago inaequilateralis*, and *Ficus microcarpa*) were presented in the list of the top 10 food species during both study periods (Table 5). Of these, *P*. *tatarinowii* was consumed in large quantities in both years and consumed every month during the recording period. The main parts of *P*. *tatarinowii* were young leaves and mature leaves. These species were the main food sources of the langurs.

**TABLE 5:** The top 10 plant species consumed by white‐headed langurs in 2013 and 2016 in Chongzuo White‐headed Langur National Nature Reserve

Species	Family	Life form	2013			2016		
			Plant parts	No. of months used	% (*F*)^b^	Plant parts	No. of months used	% (*F*)
*Pteroceltis tatarinowii*	Ulmaceae	Tree	YL, ML	11	14.8	YL, ML	11	18.0
*Cuscuta chinensis*	Convolvulaceae	Vine	FL, ST	11	8.4			
*Canthium dicoccum*	Rubiaceae	Tree	YL, ML, FR, FL	11	8.2	YL, ML, FR, FL	7	3.6
*Cansjera rheedei*	Opiliaceae	Vine	YL, ML, FR, S, ST	11	5.7	YL, ML, FR	11	7.0
*Sterculia monosperma*	Sterculiaceae	Tree	YL, FR	11	4.4	YL, FL,FR,S	7	4.1
*Ventilago inaequilateralis*	Rhamnaceae	Vine	YL, FR	11	3.9	YL, FR, FL,	8	5.7
*Sinosideroxylon pedunculatum*	Sapotaceae	Tree	YL, ML, FR	8	3.6			
*Ficus microcarpa*	Moraceae	Tree	YL, FR	10	3.5	YL, FR	8	3.2
*Gymnema sylvestre*	Asclepiadaceae	Vine	YL, ML, FR, FL	11	3.1			
*Broussonetia papyrifera*	Moraceae	Tree	YL, ML, FR, FL	9	2.5	YL, ML, FR, FL	8	
*Iodes vitiginea*	Icacinaceae	Vine				YL, ML, FR, FL	10	7.8
*Vitex tripinnata*	Verbenaceae	Tree				YL, FR	8	5.9
*Bauhinia championii*	Caesalpiniaceae	Vine				YL, S	7	4.8
*Apodytes dimidiata*	Icacinaceae	Tree				YL, ML, FR, FL	11	4.0
Sum of top 10 food species					58.1			64.3

^a^ Plant part: YL, young leaf; ML, mature leaf; FR, fruit; S, seed; FL, flower; ST, stem.

^b^ %(F): percentage of total feeding record.

## 4 DISCUSSION

This study focuses on the feeding behavior of white‐headed langurs and analyzes their dietary composition and proportion, the relationship with food availability, and dietary diversity and variations. Further, we discuss the causes of food choices and the seasonal and interannual variations, which could be helpful in understanding the significance of these strategies for white‐headed langurs adapting to fragmented limestone forests.

White‐headed langurs are typical limestone forest folivores, and young leaves are their first choice of food items (Li & Rogers, 2006; Yin et al., 2011; Zhou et al., 2013). In this study, leaves were the main food item of the langurs during the two study periods. However, the consumption of leaves showed significant seasonal differences within a year and across years (Tables 1 and 2). This result is similar to that of previous reports on white‐headed langurs (Huang, Wu, et al., 2008; Li & Rogers, 2006; Yin et al., 2011). Meanwhile, the consumption of young leaves was confirmed to correlate positively with their availability, which is similar to other studies showing that the consumption of staple foods varies with the seasonal variations in its availability (Chaves et al., 2012; Tsuji et al., 2006).

Most wild primates tend to choose food that is easily available, thus conserving energy (Richard, 1985). In this study, however, even though mature leaves were abundant and essentially available, they did not consume them as a staple food. Mature leaves are used as fallback food when young leaves are lacking, because mature leaves are nutrient‐rich and rich in fiber (Li et al., 2013; Oftedal, 1991; Richard, 1985). The same results were obtained in previous studies of white‐headed langurs (Huang, Wu, et al., 2008; Li & Rogers, 2006; Zhou et al., 2013) and other limestone‐dwelling primates (Huang et al., 2010; Tang et al., 2016). François’ langurs are typical folivores that mostly consume young leaves and supplementary mature leaves in the dry season (Hu, 2011; Huang et al., 2010; Zhou et al., 2009). Similarly, rhesus macaques that live in limestone forests are folivores that have a priority selection for young leaves; they barely feed on mature leaves in the rainy season when young leaves are abundant (Tang et al., 2016). Thus, primates inhabiting limestone forests tend to use mature leaves as their fallback food, which is a response to the seasonal variation of food resources in the limestone forests, as well as an effective strategy for adapting to their unique habitat.

Many researchers have reported that primates increase their consumption of seeds, stems, and petioles to supplement energy when their main sources of food are lacking (Amato et al., 2015; Hu, 2011; Huang, Wu, et al., 2008; Huang et al., 2010). For instance, François’ langurs have been found to increase their consumption of stems and seeds during the dry season (Huang et al., 2010; Tang et al., 2016; Zhou et al., 2009). In this study, the consumption of seeds significantly increased during the dry season of 2016. Studies have shown that seed yields are susceptible to climate change and show significant interannual variability (Zhou et al., 2009). Seeds are important supplementary foods when young leaves are scarce and are an important energy source for primates as they are rich in fats and starch (Richard, 1985). In addition, the consumption of stems and petioles also significantly increased in the dry season of 2013, which may be related to rainfall. Rainfall has a strong influence on food availability, such as young leaves and fruits (Huang et al., 2015), among which young leaves are the water source for these langurs (Huang, 2002). Rainfall was lower in 2013, probably resulting in more water supplement pressure during this period. The stems and petioles have relatively higher moisture content (Waterman & Kool, 1994); therefore, a higher consumption of these items in the dry season of 2013 could be because they were a better choice for water replenishment during such periods of low rainfall.

In general, food diversity is influenced by feeding habits and food availability; for example, when wild animals spend most of their time on several staple food species, the diversity of their diet is reduced (Bennett, 1986; Fleagle, 1984; Li et al., 2003, 2009). In this study, the data from 2016 showed that the diversity of the diets of the langurs decreased when they increased their consumption of young leaves and increased when the consumption of mature leaves was increased. This indicates that during the rainy season, the langurs fed on several plants with abundant young leaves, such as *P*. *tatarinowii*, *S*. *monosperma*, and *F*. *microcarpa* (Table 5). Similarly, research on François’ langurs also showed a negative correlation between diet diversity and young leaf consumption (Li et al., 2008). Compared with frugivores, the folivore primates were found to have a lower diet diversity, with the diversity indices of frugivorous *Lemur* *catta* and *Propithecus diadema perrieri* ranging ~6.37–6.51 and ~6.12–6.35, respectively (Lehman & Mayor, 2004), which is significantly higher than that of the white‐headed langurs (~2.67–4.39; in this study) and François’ langurs (~1.76–2.53) (Li et al., 2008). Furthermore, fruit‐preferring macaques in limestone forests have also shown similarly low levels of diet diversity as langurs, as reported in rhesus macaques (an average of 2.408) and Assamese macaques (an average of 1.164) (Zhou et al., 2014). These studies may reflect a unifying trend that limestone‐dwelling primates are predominantly characterized by a low diet diversity that varies with food availability (this study; Li et al., 2008; Zhou et al., 2014). Habitat fragmentation interrupts interactions between species and has a strong negative impact on biodiversity, which may lead to a decrease in plant diversity and their progeny quality in small patches (Aguilar et al., 2019). Thus, the lower diet diversity of limestone‐dwelling primates may be the result of their adaptation to fragmented limestone forests (Li et al., 2008, 2015).

The number of plant species consumed and their diversity were significantly different between the two study years; both were higher in 2013 than in 2016. François’ langurs living in the Nonggang Nature Reserve have also shown significant interannual variations in the consumption of plant species (Zhou et al., 2009). This is likely to be because of the differences in rainfall and phenology between the two study periods. Rainfall was significantly higher in 2013 than in 2016, which favored the growth of plants, perhaps providing more diverse food resources for the langurs. This is supported by the fact that the availability of young leaves was higher in 2013. Thus, the number and diversity of food species varied with the phenology of different years, reflecting their flexible food choice strategies.

## 5 CONCLUSION

In summary, our results confirm that the consumption of young leaves in white‐headed langurs varied with the availability of young leaves, wherein mature leaves were a supplementary food consumed during the dry season when young leaves were lacking. The decrease in diet diversity during the rainy season may be related to the increase in the consumption of young leaves. There were significant interannual variations in the consumption of plant species in the langur diet, which was probably related to the variations in phenology. These results not only contribute to understanding the feeding ecology of limestone‐dwelling primates, but also have important implications for the conservation and management of white‐headed langurs.

## CONFLICT OF INTEREST

None declared.

## AUTHOR CONTRIBUTION

**Shiyi Lu:** Investigation (equal); Writing‐original draft (equal). **Ting Chen:** Data curation (equal); Investigation (equal); Writing‐original draft (equal). **Zhonghao Huang:** Methodology (equal); Writing‐review & editing (equal). **Youbang Li:** Funding acquisition (equal); Supervision (equal). **Changhu Lu:** Formal analysis (equal); Methodology (equal); Project administration (equal); Supervision (equal).

## Data Availability

All data are available in the figshare repository at https://doi. org/10.6084/m9.figshare.12979889.v1.
